# How Older Female Spouses Cope with Partners' Coronary Artery Bypass Graft Surgery

**DOI:** 10.1155/2013/923137

**Published:** 2013-03-24

**Authors:** Suzanne Marnocha, Mark Marnocha

**Affiliations:** ^1^College of Nursing, University of Wisconsin Oshkosh, Oshkosh, WI 54901-8660, USA; ^2^Department of Family Medicine, University of Wisconsin School of Medicine and Public Health, Fox Valley Campus, Appleton, WI 54911-5725, USA

## Abstract

This research sought to better understand how older female spouses cope with a partner's coronary artery bypass graft surgery and to explore coping's relationships with life-change stress, cognitive appraisal, resilience, social support, and aspects of spouse's surgery. A sample of 96 women, aged from 55 to 81 years, completed surveys after their partner's surgery. Folkman and Lazarus' ways of coping (WCQ) scales yielded two factors in this sample—reactive coping and adaptive coping. Reactive coping, including more emotion-focused ways of coping from the WCQ, was associated only with more time spent anticipating spouses' surgeries. Women described the greatest use of ways of coping labeled adaptive, which in turn had significant relationships with greater resilience, social support, and positive appraisal of the surgical experience. Stepwise multiple regression found greater resilience, more frequent religious participation, and fewer children to be distinct predictors of adaptive coping. Nursing staff are encouraged to accept and normalize reactive coping, while facilitating adaptive coping with surgical stresses.

## 1. Introduction

The leading cause of noncommunicable death (NCD) world-wide in 2008 was cardiovascular disease, accounting for 17 million, or 48%, of all NCD deaths [1]. Heart disease may lead to patients' physical and emotional distress, job loss, disability, and reduced quality of life [2–4]. While much empirical research suggests that coronary artery bypass graft (CABG) surgery is stressful for the patient [5], the patient's spouse may be under more stress than the patient [6, 7]. The spouse may demonstrate poorer psychological adjustment and higher levels of anxiety and depression than the patient [6], both immediately and following acute cardiac events, such as acute myocardial infarction (AMI), heart failure [8], CABG surgery [2], as well as during the first 3 months after hospital discharge [9, 10]. During and after acute cardiac interventions, spouses may well be “forgotten” in an environment devoted to the patient [11–13]. Spouses may also be more immediately concerned about the changing marital roles, as the burden of care giving and other responsibilities falls on their shoulders [6, 7].

While the impact of partners' CABG surgery upon spouses is well documented in general, there is a dearth of research concerning how the older female spouse copes with her partner's acute surgical event. Artinian's research [11, 12, 14], along with Dracup et al.'s work [8], has addressed multiple factors that may affect the spouse's coping with a partner's cardiac event, such as cognitive appraisal of the event's impact, personal resilience, amount of social support, and recent family life-change stress. Research that furthers understanding of how older female spouses cope with a partner's CABG surgery may contribute to spouses' welfare, as well as to improved outcomes for partners [6].

Lazarus and Folkman's [15] theory of stress has been applied in diverse healthcare research settings and continues to influence the most current theory and research on coping. The authors' [15] contextual and cognitive model suggests that coping focused on problem resolution is more adaptive than coping focused on emotion. However, the dynamic aspects of their theory imply that differing coping styles may be appropriate depending on whether utilized at the onset of a stressful event or later, and upon the person's appraisal of the situation and of their resources. When faced with acute stresses with little perceived control over outcomes and high levels of uncertainty, emotion-focused coping may be more effective, allowing reductions in distress and increases in support. The initial reactions to a partner's impending CABG surgery may be most characterized by emotional coping [15, 16], to include distancing, self-control of emotion, escape/avoidance, accepting responsibility, and confrontive coping. However, successful adaptation post-surgically requires active problem-focused coping efforts, as well. Folkman and Moskowitz [17] reviewed diverse research suggesting that social support coping is not just emotion focused, but has an important problem-solving component, as does positive reappraisal coping. Such problem-focused coping, broadly defined to include positive reappraisal, meaning-focused, and social support aspects, may be difficult for older female spouses in a medical environment that may encourage passivity and escape or avoidance patterns. Subsequent analyses of coping have suggested another distinction, that between disengagement coping reactive to a stressor and engagement coping via initiation of adaptive problem-solving [18–20]. Thus, it may be useful to explore what are the characteristics of spouses and the situations associated with use of reactive emotion-focused coping or adaptive problem-focused coping, and whether there are differences in the use of coping associated with age variations among older female spouses.

Wagnild and Young's Resilience Scale (RS) [21] was developed to assess five themes found in interviews with older women who had adapted successfully to a major life event: (a) equanimity, (b) perseverance, (c) self-reliance, (d) meaningfulness, and (e) existential aloneness. The RS has shown validity in several studies of older women adjusting to loss [22, 23]. While resilience is sometimes treated as if it was a personality trait, its original definition suggests it is composed of specific skills and strategies which can be taught and strengthened [22]. The resilient woman's emotional balance, healthy independence, and ability to find positive meaning in stressful circumstances would likely facilitate higher levels of reported problem-focused coping, as well as adaptive flexibility.

Spouses' perceptions of life-change stress and social support may affect emotional distress and, therefore, ways of coping [14]. Women who report more life-change stress or less social support may have difficulty mobilizing adaptive problem-focused coping due to greater perceived situational demands and less perceived personal resources. These women may spend more effort on reactive emotion-focused coping in order to achieve emotional balance and favorable perceptions. 

This study aims to describe patterns of coping among older female spouses dealing with a partner's CABG surgery and the relationships of these patterns with life-change stress, cognitive appraisal of CABG surgery, resilience, social support, demographic factors, and aspects of the surgery. Based on these theoretical frameworks and prior research [14, 24], it is hypothesized that women who are resilient and report higher levels of social support, fewer competing life stresses, and more favorable appraisal of the stressful circumstance of CABG will report greater use of adaptive problem-focused coping with a spouse's CABG event and less use of reactive emotion-focused coping.

## 2. Materials and Methods

### 2.1. Design

A retrospective, descriptive, and exploratory survey design was used, with questionnaires distributed to a convenience sample of older female spouses of male post-CABG partners. The study was reviewed and approved as exempt by the appropriate hospital and university Institutional Review Boards.

### 2.2. Setting and Sample

Ten clinical sites were recruited from two midwestern hospital systems in the USA. Sites included two cardiovascular surgery offices, three cardiac rehabilitation facilities, two surgical preteaching departments, and three post-surgical units. The site of origin of surveys was not recorded. Site staff distributed survey packets to female spouses of patients who had undergone CABG within the previous 3 months. All eligible spouses, those of 55 years of age or older, and able to speak, read, and understand English, were provided an informational letter, along with the survey packet, which explained the study and its anonymous nature. Consent was presumed if the spouse mailed back the survey packet in the stamped and addressed envelope provided with the packet. A total of 106 surveys were distributed by research sites, and 96 usable surveys were completed and returned, yielding a 91% response rate. Due to the anonymous nature of the study, there is no identifying personal data for those who completed the survey nor is there any demographic data for those who declined.

### 2.3. Measures

Demographic information included participant's age, ethnicity, religious activities, educational level, number of years married, number and location of children, employment status, family income, comprehensive health insurance, personal illnesses in the past year, CABG aspects of timing and presurgical education, financial strain, and partner's cardiac history. Life-change stress was evaluated using the Family Inventory of Life Events (FILE), a measure of how many diverse family events and changes have occurred during the previous year [14, 25]. For this sample, a FILE item asking about respondents' difficult pregnancy in the past year was omitted. Cognitive appraisal of partner's surgery was measured by the validated spouse perception scale (SPS) [26, 27]. The SPS provides a total score reflecting how favorable spouses' attitudes are toward their partner's recent surgery. Resilience was measured by total score on the Resilience Scale (RS) [21, 23]. The social support index (SSI) yields a total score measuring respondents' evaluation of family integration and support in the community [28, 29]. Reliabilities observed for all these measures in the present sample were acceptable (Cronbach's alphas .78 to .94) and comparable to values reported in prior studies noted above.

The ways of coping questionnaire (WCQ) [16] asked spouses to respond according to how they coped with their partners' CABG, during and since surgery. The WCQ provides average scores (ranges from 0 to 3, corresponding from “not used” to “used a great deal”) for each of eight coping scales. In this sample, reliabilities of these coping scales were acceptable for research purposes (Cronbach's alphas .49 to .72). Folkman and Lazarus [16] have indicated that reliabilities may trend lower for these scales because a few items within a coping scale may be very highly rated, while others are rated minimally or not at all.

Folkman and Lazarus provide succinct descriptions of the content of the coping scales, which have been validated in research on older adults, as well as caregivers and family members of those with acute or chronic illness [30–33]. Planful problem solving refers to deliberate problem-focused efforts, while seeking social support represents the pursuit of tangible, informational, and/or emotional support from others. Positive reappraisal refers to efforts to create positive meaning and to use a religious dimension in coping, while self-control coping involves efforts to regulate or moderate one's feelings and actions. Distancing coping involves cognitive efforts to detach from or minimize the significance of the stressful situation, while escape-avoidance refers to wishful thinking and behavioral escape from problem circumstances. Confrontive coping takes the form of aggressive action and risk-taking in response to stress, while accepting responsibility involves a focus upon one's own role or responsibility and efforts to atone or make things right [16]. Both confrontive and accepting responsibility scales include items pertaining to strong emotions of anger or guilt. Recent work has resulted in multiple reanalyses and rescoring of the WCQ, and previous authors suggest that the WCQ's structure should be reexamined for populations under investigation [20, 34]. Therefore, the WCQ scales were factor analyzed in the current sample. 

### 2.4. Data Analysis

Relationships among descriptive and demographic variables were examined depending on scales of measurement, via either Chi-squared analyses or one-way ANOVAs and independent sample *t*-tests (all with *P* < .05, 2-tailed). A MANOVA was conducted to assess differences in WCQ scores as a function of spouse age groups. Paired *t*-tests were used to ascertain significant differences among coping scores in the overall sample. In order to further describe and simplify broad patterns of coping in this particular sample, exploratory factor analyses of the eight coping subscales were conducted using principal components factoring and Varimax rotation. Relationships among coping factors and the predictor variables noted (FILE, SPS, SSI, RS) were assessed via 2-tailed bivariate Pearson correlations (*n* = 96). Pilot regression analyses were conducted to ascertain sets of variables predictive of ways of coping, as measured by factor scores. An original power analysis calculation determined a sample size of 61 would be sufficient to detect a Pearson correlation of .35 (2-tailed alpha of <.05) with a power of .80 [35–37]. In light of the descriptive and exploratory nature of this research, power was based on simple correlations, and regression analyses were considered exploratory and subject to replication. Calculations and statistical procedures were conducted using SPSS version 11.5.0.

## 3. Results

These 96 female spouses of CABG patients were mostly European-American, unemployed, of modest income, and at least high school educated, with over 25% reporting some college-level education. Marital relationships were long term, and most spouses had at least one child living nearby. Mean age was 65.8 years, with a range from 55 to 81 and only 7 spouses over 75 years of age. The sample showed approximately 33% either older than 70 years, 62 to 70 years, or younger than 62 years, and these cutoffs were used in subsequent analyses in order to maintain similar numbers of subjects among the age groups. Most spouses denied financial hardship due to CABG, reported comprehensive health insurance, noted relatively few illnesses of their own in the past year, and reported high levels of religious involvement. Most spouses reported that partners had not experienced a prior myocardial infarction or CABG surgery. Approximately 50% of spouses reported one week or more lead time between being informed of the need for surgery and the surgery itself, while 30% of spouses reported urgent/emergent surgeries, those with a day or less elapsing between identified need and the surgery itself. Survey data did not include clinical rationales for variations in surgery lead time. Nearly 50% of spouses reported pre-surgical education.  Table 1 presents demographic data and Table 2 presents stress, coping, and personality data for the sample.

Age, analyzed for the three age groups noted above (over 70 years, 62 to 70 years, under 62 years), showed significant relationships with employment status, household income, and religious participation. Oldest women, compared to the two younger groups, reported much less employment, part or full-time (6%, 32%, and 77% employment, respectively; chi-square  (4 df) = 30.6, *P* < .001) and higher rates of at least weekly church attendance (97%, 68%, and 60%, respectively; chi-square  (10 df) = 22.9, *P* < .05). The older groups, compared to the youngest, reported lower average household income ($28,971, $34,677, and $51,452, respectively; *F*(1,93) = 10.78, *P* < .001). Age was not related to reported comprehensive health insurance coverage or financial strain.

Women reported significant differences among means for the eight WCQ ways of coping scales, which are presented in Table 2, from most used to least. Paired-observation *t*-tests indicated statistically significant differences (*P* < .02) for all pairs of WCQ scales, with the exception of differences between confrontive and escape-avoidance coping, and between seeking social support and planful problem-solving coping. The means for positive reappraisal, planful problem-solving, and seeking social support were notably higher than the means for other ways of coping, while confrontive, escape-avoidance and accepting responsibility coping were notably lower, with means below .5 on the 3-point rating scale. The WCQ scale scores did not vary significantly among age groups, nor did they show any significant correlations with actual age.

An exploratory factor analysis on the WCQ coping subscales extracted two factors with eigenvalues >1.0, accounting for 70.6% of total variance. The first, accounting for 57.4% of total variance, had rotated loadings greater than .74 for escape/avoidance, confrontive, accepting responsibility, self-controlling, and distancing coping. This factor was termed reactive coping based on its mixture of strong emotional reactions turned inward (guilt) or outward (anger) coupled with disengagement coping, such as escape/avoidance or suppressed responding. The second factor, accounting for 13.2% of total variance, had loadings greater than .64 for seeking social support, positive reappraisal, and planful-problem solving coping. This factor was termed adaptive coping based on its loadings for positive reappraisal, seeking of social support, and planful problem-solving, all suggesting an engaged instrumental approach to the CABG experience. Factor scores were calculated and used in subsequent analyses.

Life-change stress (FILE), spouse appraisal of the CABG experience (SPS), resilience (RS), and social support (SSI) were all significantly correlated. Spouses with more life-change stress showed lower resilience scores, less perceived social support, and less favorable appraisal of the CABG experience. Resilience showed positive associations with perceived social support and favorable appraisal of the CABG surgery. Perceived social support and favorable appraisal were positively correlated. Reactive coping showed no significant correlations with resilience, social support, life stress, or favorable appraisal of the surgical experience. However, adaptive coping was associated with greater resilience, more social support, and more favorable appraisal.  Table 3 presents correlations and significance levels.

Stepwise multiple regressions then examined best predictors of reactive and adaptive coping. Variables entered into each analysis included FILE, RS, SPS, SSI, demographic measures, time elapsed between CABG and survey completion, time between acute diagnosis and surgery, participation in pre-surgical education, spouses' illnesses in the past year, and spouses' religious participation. Demographic measures included age, years of marriage, educational level, income, number of children, and number of children living nearby. Stepwise regression for reactive coping yielded a single predictor, time between acute diagnosis and CABG surgery, with an adjusted *R* ^2^ of .050 (*F*(1,93) = 5.92, *P* < .05) and a standardized Beta of .245 for the single predictor. Having spent more time anticipating the surgery predicted greater reported use of reactive coping. Stepwise regression for adaptive coping yielded three predictors in the final equation, with an adjusted *R* ^2^ of .202 (*F*(3,91) = 8.94, *P* < .001). Fewer children (standardized Beta = −.343), more frequent religious participation (standardized Beta = .238), and greater resilience (standardized Beta = .314) were predictive of greater use of adaptive coping.

## 4. Discussion

As expected from the theoretical frameworks and conceptual definitions, spouses with more resilience reported more social support, more positive appraisal of the surgical experience, more use of adaptive coping, and less life-change stress. Contrary to expectation, more emotion-focused coping, as measured by the reactive coping factor and WCQ subscales, showed minimal relationship with resilience, social support, or life-change stress. The spouses demonstrated a distinct profile of coping, reporting the least use of ways of coping within the reactive coping factor and the most use of ways of coping within the adaptive coping factor. These coping factors are not identical to the emotion-focused versus problem-focused distinction, but are consistent with Folkman and Moskowitz's [17] review of coping and other recent reviews of the coping literature [18]. As Lazarus and Folkman [15] have suggested, and has recurred in writings and research since [17–19], coping is indeed a flexible process, wherein all these ways of coping have healthy roles, as well as less healthy ones.

 This view of spouses' coping is supported by regression analyses for each coping factor. The only significant predictor of reactive coping was more time reported between diagnosis and surgery. This result suggests that more time spent in a holding pattern, uncertain about what could be done to resolve problems, is associated with greater use of reactive coping. In contrast, adaptive coping was strongly associated with resilience. Having more children predicted less adaptive coping, perhaps due to how a mixture of social demands and social support provided by a larger family may supplant intentional adaptive coping efforts. Finally, as suggested by Folkman and Moskowitz's [17] review of coping and the contributions of such researchers as Pargament [38], amount of religious participation was predictive of more use of adaptive coping. 

This study is limited by its retrospective approach, though Folkman and Moskowitz [17] suggest that such an approach may allow more understanding of the spouses' narratives of how they perceived and went through the stressful events. This study did not have measures of mental health outcomes, such as anxiety or depression, though the dynamic nature of coping suggests that such mental health issues may guide the next round of coping processes rather than representing fixed endpoints. Nonrandom sampling poses a greater challenge, in that those sampled may have been better known by staff and had partners more involved in cardiac followup. In addition, this sample may have excluded those with surgical experiences that were devastating, or those most noncompliant with or alienated from the healthcare system. In particular, those with spouses who did not survive the acute cardiac events were still receiving acute care for other emergent medical problems or were transferred to residential care would be less likely to have been recruited for this study. Measures within the study did not include any objective clinical data on how complex and difficult the CABG experiences were. The current sample did not have a good representation of the oldest-old group of aging women, those 80 or older [39], with only a small percentage of spouses older than 75. The projected growth of that segment of the older female population in future decades suggests an ongoing need for further research on how the most senior women successfully cope with critical family illnesses.

These results suggest that care for older female spouses after partners' CABG surgery needs to address life event stresses arising from the surgery itself or from other life areas, to educate the spouses concerning social support resources, to facilitate assertive problem-solving, and to suggest resources for rehabilitation and wellness that may facilitate positive reappraisal and a sense of meaning. Nursing staff should accept and validate expressions of reactive coping during the initial diagnosis and during circumstances that force families to cope with delay or uncertainty. Spouses who use the extremes of reactive coping may take on undue guilt or responsibility, may more aggressively confront the healthcare system, may suppress or deny emotions more rigidly, and may show avoidance of the surgical situation. These patterns are part of reactive coping and do not limit or preclude successful use of adaptive coping. Linnarsson et al. [40] have conducted a metasynthesis of qualitative research on the needs and experiences of critically ill or injured patients' significant others (SO), defined to include “all persons close and significant to the patient…” [40]. They noted within diverse studies five content themes, one of which (uncertainty and emotional “roller coaster”) captures the sense of reactive coping: an “initial chaotic time and the uncertainty led to a strong feeling of being powerless and not being able to do anything to help … keeping it together trying to avoid breaking down with emotion, acting out … some talked about distancing themselves to bear the pain” (3104). Content within their other four themes aligned with aspects of resilience, social support, and adaptive coping: (a) balancing hope and reality, (b) protecting and guarding the loved one, (c) alliance with caregivers, and (d) social network support. The adaptive versus reactive coping dimensions in the current research thus appear parallel to the experiences of diverse significant others close to a critically ill person. 

Adaptive coping can be supported by nursing interventions that include mobilizing specific resources for problem-solving, encouragement of social support in order to maintain emotional stamina, and careful attention to the spouse's efforts to reappraise and find meaning in an extremely demanding experience. Qualities of resilience overlap with components of adaptive coping and together suggest a set of skills and strengths that warrant attention during and after the surgical experience. Staff may do well to accept and normalize reactive coping, even in its extreme forms, while providing opportunities, encouragement, and support for spouses' use of adaptive coping skills. As Linarrson et al. [40] observe, “significant others face an overwhelming and emotionally challenging situation, and they need to be seen and heard by the caregivers” (3109-3110). Staff may encourage resilience and adaptive coping via uncovering spouses' previous successes coping with extreme disruption, mobilizing use of religious resources, and supporting qualities of emotional stamina, courage, and adaptability in stressful circumstances [22, 23].

## Figures and Tables

**Table 1:** Spouses' demographics (*n* = 96).

Demographics	Mean	SD	Number	Percentage
Age	65.8	7.2		
<62 years			31	32.3
62 to 70 years			31	32.3
>70 years			34	35.4
Income				
<$20,000			16	16.6
$20,000–$29,999			31	32.3
$30,000–$49,999			26	27.1
>$49,999			23	24.0
Race/ethnicity				
European-American			96	100.0
Employment status				
Full time			15	15.6
Part time			15	15.6
Not employed			52	54.2
Item omitted			14	14.6
Educational level				
High school or less			74	77.0
Some college			11	11.5
College degree or above			11	11.5
Children				
Yes			92	95.8
No			4	4.2
How many children	3.7	2.9		
Children nearby	2.4	2.0		
Years of marriage	40.5	13.4		
Comprehensive health insurance				
Yes			91	100.0
Missing data			5	
Financial hardship due to CABG				
Yes			34	35.4
No			62	64.6
Religious attendance Frequency				
Daily			18	18.7
Weekly			55	57.3
Monthly or less			23	24.0
Presurgical education				
Yes			45	46.9
No			51	53.1
Number of illnesses in the past year	1.4	1.3		
Days waiting for CABG				
Less than 1 day			28	29.5
1 day to 1 week			20	21.0
>1 week			47	49.5
Missing data			1	

**Table 2:** Stress, personality, and coping data.

	Mean	SD	Range
Stress and personality measures			
FILE (life-change stress)	5.4	4.6	0–20
Spouse's perception scale (appraisal)	178.9	21.3	122–229
Resilience	139.4	20.7	54–175
Social support index	51.2	8.4	27–68
Ways of coping (WCQ raw scores)			
Positive reappraisal coping	1.35	.59	.14–3.00
Planful coping	1.19	.60	.17–2.67
Seeking social support coping	1.19	.58	.00–2.50
Self-controlling coping	1.02	.59	.00–2.71
Distancing coping	.74	.46	.00–2.17
Confrontive coping	.48	.45	.00–2.33
Escape-avoidance coping	.46	.35	.00–1.38
Accepting responsibility coping	.33	.53	.00–2.25

**Table 3:** Correlations among stress and coping measures.

	CABG appraisal	Resilience	Social support	Reactive coping	Adaptive coping
Life-change stress	−.38***	−.37***	−.34***	.01	.02
CABG appraisal		.46***	.35***	.00	.21*
Resilience			.39***	−.05	.26**
Social support				−.06	.20*

All values are 2-tailed Pearson correlations (*n* = 96).

*P < .05.

**P < .01.

***P < .001.
